# The P2X7 Receptor and Its Relation to Neglected Tropical Diseases: Focusing on Chagas Disease

**DOI:** 10.1155/jotm/7015795

**Published:** 2026-01-21

**Authors:** Caroline de Souza Ferreira Pereira, Robson Xavier Faria

**Affiliations:** ^1^ Laboratory of Evaluation and Promotion of Environmental Health, Fiocruz, Oswaldo Cruz Institute (IOC), Avenida Brasil 4365, Rio de Janeiro, CEP 21040-900, Brazil, fiocruz.br

**Keywords:** dengue, leishmaniasis, leprosy, neglected tropical diseases, purinergic signaling, schistosomiasis, *Trypanosoma cruzi*

## Abstract

Chagas disease, caused by *Trypanosoma cruzi*, is a neglected tropical disease (NTD) that can lead to severe cardiac complications, including chronic Chagas cardiomyopathy. While NTDs are caused by a variety of pathogens—such as protozoa, bacteria, viruses, and helminths, Chagas disease remains underexplored, particularly regarding host immune responses. In this context, purinergic signaling has gained attention as a relevant pathway in the regulation of both infection and inflammation. Extracellular adenosine triphosphate (ATP), commonly elevated during inflammatory conditions, acts through P2 receptors, with P2X7 standing out for its ability to induce cell death and modulate cytokine release. This study investigates the involvement of the P2X7 receptor in NTDs, with a particular focus on Chagas disease, due to its established association with cardiovascular inflammation and its potential role in *T. cruzi* infection. Although other NTDs were initially considered, some NTDs were not investigated in detail because of insufficient data linking P2X7 receptor activity to their pathogenesis. Consequently, the analysis concentrated on Chagas disease, where current evidence indicates that P2X7 receptor activation increases proinflammatory cytokine levels and may contribute to disease progression, especially in its cardiac form. Thus, P2X7R emerges as a promising molecular target for therapeutic strategies and may serve as a potential biomarker for identifying early or indeterminate forms of Chagas disease.

## 1. Introduction

The P2X7 receptor is a ligand‐gated ion channel and a member of the purinergic receptor family, playing a pivotal role in cellular signaling pathways [1]. This receptor is broadly expressed across various cell types and tissues, including blood cells, neural cells, stem cells, and muscle cells. Activation of the P2X7 receptor triggers a wide array of cellular responses, most notably the release of inflammatory mediators, such as proinflammatory cytokines. Among these, interleukin‐1β (IL‐1β) is a key effector molecule whose maturation and secretion are dependent on inflammasome activation, thereby underscoring the receptor’s central role in orchestrating inflammatory processes [2, 3].

The functional consequences of P2X7 receptor activation are highly context‐dependent, capable of eliciting either protective or deleterious effects depending on the cellular environment and activation state. The capacity of pathogens to invade host cells and modulate P2X7 expression is influenced by the specific cell type involved. Furthermore, the activation of this receptor has been implicated in the pathogenesis and immune regulation of numerous infectious diseases caused by viruses, bacteria, fungi, protozoa, helminths, and other pathogenic organisms [4].

Neglected tropical diseases (NTDs) predominantly afflict socioeconomically disadvantaged populations residing in low‐income countries, particularly within rural regions. Representative examples of NTDs include Chagas disease, leprosy, schistosomiasis, leishmaniasis, and dengue. These diseases are responsible for significant morbidity, disability, and mortality, posing serious public health challenges in endemic regions [5]. The continuous emergence and re‐emergence of pathogens with novel or enhanced virulence factors exacerbate the global burden of these diseases, resulting in millions of deaths annually. A multifactorial interplay of microbial, political, environmental, human, technological, social, and ecological determinants drives the persistence and proliferation of NTDs [6].

Despite the pressing need for novel therapeutic interventions, progress in drug development for NTDs has been remarkably limited. Between 1975 and 2004, out of 1556 newly approved pharmaceutical agents, a mere 1.3% (21 drugs) were designated for the treatment of NTDs. This striking disparity underscores the critical necessity of identifying new molecular targets and advancing research aimed at developing effective therapies for these neglected diseases [5].

## 2. Purinergic Signaling: Focus on the P2X7 Receptor

Adenosine triphosphate (ATP) is a fundamental source of cellular energy produced by organisms and utilized by various cell types. Although ATP is predominantly confined to the intracellular compartment under physiological conditions, it can also be released into the extracellular space, where it exerts signaling functions by interacting with membrane‐bound purinergic receptors [7]. Extracellular ATP (eATP) serves as a key mediator in purinergic signaling pathways, which regulate a wide spectrum of physiological and pathological processes, including inflammatory responses [7].

The regulatory mechanisms of eATP involve four principal processes: (1) binding of ATP to specific purinergic receptors (P2Rs), thereby triggering metabotropic or ionotropic cellular responses; (2) passive release of intracellular ATP through cell lysis or active release mediated by membrane transport proteins; (3) enzymatic hydrolysis of eATP by membrane‐bound ectonucleotidases; and (4) subsequent dephosphorylation of eATP into nucleosides, which are taken up by cells through salvage pathways. Infections caused by protozoan parasites can disrupt these homeostatic mechanisms, altering eATP concentrations [8].

Multiple cell types release ATP and other nucleotides, including ADP, UTP, and UDP, in response to apoptosis, mechanical injury, necrosis, or various chemical and mechanical stimuli. The P2X7 receptor, upon activation, facilitates ATP permeation through the formation of transmembrane pores, either independently or via interaction with pannexin‐1 hemichannels [8, 9]. ATP was initially identified as a cotransmitter in both parasympathetic and sympathetic neurons. Purinergic receptor subtypes are widely distributed in neurons and glial cells within the central nervous system (CNS) [10].

Adenosine acts on four distinct P1 purinergic receptors (A1, A2A, A2B, and A3), all of which are G protein–coupled receptors (GPCRs). Typically, A2A and A2B receptors stimulate cyclic adenosine monophosphate (cAMP) production via Gs proteins, whereas A1 and A3 receptors inhibit cAMP synthesis through Gi/o proteins [10]. Adenosine modulates various neural and glial functions, including neuronal development and neuron–glia communication. Dysregulation of adenosine signaling has been implicated in psychiatric disorders and neurodegenerative diseases. Furthermore, adenosine may exert synergistic or antagonistic effects on ATP‐mediated responses, depending on the context [10].

The purinergic signaling cascade initiates with the release of ATP from the intracellular compartment into the extracellular environment. Both ATP and ADP interact with P2 purinergic receptors, which are classified into two families: P2Y (metabotropic) and P2X (ionotropic) receptors [11]. P2Y receptors, as members of the GPCR family, are further subdivided into two groups based on their coupling to specific G proteins. The first group includes P2Y1, P2Y2, P2Y4, P2Y6, and P2Y11, which are coupled to the Gq subunit, leading to phospholipase C‐β activation [12]. The second group comprises P2Y12, P2Y13, and P2Y14, which are coupled to Gi proteins, resulting in the inhibition of adenylyl cyclase and the regulation of ion channels. P2Y receptors are activated by a variety of nucleotides, including ATP, ADP, uridine‐5′‐triphosphate (UTP), uridine diphosphate (UDP), and inosine triphosphate (ITP) [12].

In addition to P2Y receptors, ATP activates ionotropic P2X receptors, which function as cation‐selective channels. The P2X receptor family consists of seven subtypes (P2X1 to P2X7) [3]. These receptors permit the passage of Na^+^, K^+^, and Ca^2+^ ions, thereby altering local ion gradients and membrane potential. Structurally, P2X receptors possess two transmembrane domains, with extracellular domains containing ten conserved cysteine residues that form five disulfide bridges, contributing to receptor stability and tertiary structure [8]. P2X receptors are expressed in both pre‐ and postsynaptic regions of parasympathetic and sympathetic ganglia, playing critical roles in the modulation of neurotransmitter release [10].

Among the P2X receptor subtypes, P2X7 is of particular interest due to its unique ability to form a nonselective pore upon prolonged ATP stimulation, allowing the passage of larger cations such as Na^+^, K^+^, and Ca^2+^ [13]. Activation of P2X7 receptors leads to a cascade of downstream effects, including an increase in intracellular Ca^2+^ concentrations through direct influx and mobilization from intracellular stores. This amplified Ca^2+^ response enhances the receptor’s functional output.

Furthermore, P2X7 activation promotes the assembly of the NLRP3 inflammasome complex, which is essential for the proteolytic processing and maturation of IL‐1β, a key proinflammatory cytokine. The receptor’s role in modulating membrane permeability is also notable, facilitating the efflux of K^+^ ions and enabling the permeation of large molecules either through pore dilation or interaction with secondary pore‐forming proteins. Recent studies have provided evidence that the increased membrane permeability associated with P2X7 activation is intrinsically linked to the conformational opening of its cation channel [14, 15]. These processes collectively contribute to cell death and potentiate inflammatory responses, underscoring the central role of P2X7R in immune surveillance and pathogenesis.

### 2.1. P2X7 Receptor, Inflammation, and Macrophages

The P2X7 receptor is a key modulator of cellular processes, promoting the generation of free radicals, activating phospholipases and caspases, inducing apoptosis, and regulating the cell cycle [14]. This receptor is broadly expressed across various organisms, with predominant expression in immune cells, particularly macrophages [17]. Macrophages serve as primary effectors of the innate immune response, orchestrating the migratory influx to sites of infection or injury. Upon activation, macrophages can undergo polarization into two distinct phenotypes: M1 and M2 macrophages [16, 17]. M1 macrophages exhibit a proinflammatory profile, characterized by the production of elevated levels of cytokines, reactive oxygen species (ROS), and reactive nitrogen species (RNS). Conversely, M2 macrophages are associated with immunoregulatory functions, demonstrating enhanced phagocytic activity and contributing to tissue repair and angiogenesis [18].

eATP activates the P2X7 receptor in M1 macrophages, leading to the stimulation of the NOD‐like receptor pyrin domain‐containing 3 (NLRP3) inflammasome. This activation facilitates the maturation and secretion of key proinflammatory cytokines, including IL‐1β and IL‐18. These cytokines are initially synthesized as inactive precursors (pro‐IL‐1β and pro‐IL‐18), requiring caspase‐1 (CASP1)–mediated cleavage for activation [19]. CASP1 itself is produced as an inactive zymogen (pro‐CASP1) and undergoes autocatalytic processing upon assembly within the inflammasome complex. The activation of the NLRP3 inflammasome is triggered by various stimuli, including bacterial lipopolysaccharides (LPS) and pathogen‐associated molecular patterns (PAMPs). Consequently, ATP signaling through P2X7R serves as a crucial inducer of IL‐1β secretion [19].

Interestingly, the P2X7 receptor remains functional in M2‐polarized macrophages, where it plays a role in resolving inflammation by downregulating anti‐inflammatory cytokines such as IL‐10 [16, 17]. Additionally, the P2X7 receptor is implicated in the MyD88‐dependent activation of the nuclear factor kappa B (NF‐κB) signaling pathway. MyD88, a pivotal adapter protein in LPS‐induced inflammatory signaling, regulates the transcription of various genes involved in the immune response. Experimental silencing of MyD88 has been shown to attenuate NF‐κB activation, underscoring its regulatory role [17].

In vivo studies using P2X7 receptor knockout (KO) mice have provided further insights into its immunomodulatory functions. P2X7 KO mice exhibit increased levels of IFN‐γ and IL‐10 in the footpad tissues, accompanied by heightened proliferation of CD8+ and CD4+ effector T cells. Figliuolo et al. [20] also observed a reduction in IL‐12 levels in P2X7 KO mice, potentially because of IL‐10 upregulation. Moreover, peritoneal macrophages isolated from P2X7 KO mice demonstrated impaired production of ROS and nitric oxide (NO) when compared to wild‐type controls, indicating a compromised innate immune response [20]. Collectively, these findings underscore the pivotal role of P2X7R in modulating inflammatory and infectious processes, with its effects being highly dependent on the pathogen involved and the inflammatory context.

## 3. Role of P2X7 Receptor in NTDs

The sequential dephosphorylation of ATP to adenosine follows the order: ATP > ADP > AMP > adenosine. This nucleotide degradation pathway is tightly regulated by ectonucleotidases and is directly associated with mechanisms of parasite resistance and pathogen virulence [21]. Various ectonucleotidases, including ecto‐5′‐nucleotidases, alkaline phosphatases, ectonucleoside triphosphate diphosphohydrolases (ENTPDases), and ectonucleotide pyrophosphatases or phosphodiesterases (E‐NPPs), are expressed in the brain. Among these, E‐NPPs and ENTPDases catalyze the hydrolysis of ATP and ADP to AMP, which is subsequently dephosphorylated to adenosine by ecto‐5′‐nucleotidase [10].

The role of 3′‐nucleotidases/nucleases in facilitating parasite evasion has been demonstrated in *Leishmania* infections, where these enzymes are integral to purinergic signaling pathways that promote pathogen survival. Similarly, in *Trypanosoma cruzi* infection, the activation of ectonucleotidases has been implicated in enhancing parasite persistence within the host, contributing to the establishment of chronic Chagas disease. This enzymatic activity modulates extracellular nucleotide levels, aiding in immune evasion and sustaining chronic infection [22].

### 3.1. Leishmaniasis

P2 purinergic receptors are integral to the host defense against protozoan parasites. While host organisms have evolved mechanisms to limit infection, parasites, in turn, secrete ectonucleotidases of the ENTPDase family, which neutralize P2X7 receptor‐mediated immune responses [8]. The protozoan flagellates of the genus *Leishmania*, transmitted by phlebotomine sandflies, are responsible for multiple clinical forms of leishmaniasis, including cutaneous leishmaniasis (CL), visceral leishmaniasis (VL), diffuse cutaneous leishmaniasis (DCL), and mucocutaneous leishmaniasis (MCL). CL can lead to disfiguring scars when multiple lesions are present, VL is potentially fatal if untreated, DCL results in disabling lesions, and MCL can cause severe mutilations. Approximately 350 million individuals are at risk of infection, with an estimated 500,000 VL cases and 1.0–1.5 million CL cases reported annually [23].

A study investigating localized cutaneous leishmaniasis (LCL) identified the upregulation of inflammasome‐associated genes, including P2X7, NLRP3, and IL‐1β, through immunohistochemistry, quantitative real‐time PCR, and conventional PCR assays. Elevated IL‐1β expression at both mRNA and protein levels was observed, correlating with lesion severity. The involvement of the NLRP3–ASC–CASP1 inflammasome complex in response to *Leishmania* infection has been demonstrated previously [24]. Notably, P2X7 receptor‐deficient mice exhibited increased susceptibility to *L. amazonensis* infection, characterized by larger lesions and higher parasite loads. Gupta et al. [24] also reported that IL‐1 receptor deficiency reduced edema at infection sites and conferred enhanced resistance to infection, while mice deficient in inflammasome components showed reduced neutrophil infiltration, improved parasite clearance, and lesion healing [24].

Nucleoside diphosphate kinases (NDKs) play a crucial role in maintaining intracellular nucleotide balance by transferring high‐energy phosphates between tri‐ and diphosphonucleotides. *Leishmania*‐derived NDKs contribute to parasite survival by preventing ATP‐induced cytolysis of host macrophages, thus providing a survival advantage [25].

Kushawaha et al. [25] localized *Leishmania donovani* NDKb (LdNDKb) to the parasite’s flagella, nucleus, and inner membrane. Their study demonstrated that the NDK protein from *L. amazonensis* protects macrophages from ATP‐mediated lysis by facilitating phosphate transfer and ATP sequestration. Furthermore, pretreatment with oATP, a P2X7 receptor antagonist, conferred cytoprotective effects, indicating that LdNDKb could be a potential therapeutic target [25].

Chaves et al. [26] highlighted the role of extracellular nucleotides, particularly UTP and ATP, in promoting apoptosis of *L. amazonensis*–infected cells, a process independent of NO production. Their findings also implicated LTB4 as a mediator requiring P2X7 receptor activation for effective parasite clearance, as demonstrated by reduced LTB4 release in P2X7 KO macrophages. In vivo, *L. amazonensis*‐infected P2X7‐deficient mice exhibited exacerbated lesion severity and heightened cellular infiltration at the infection site compared to wild‐type counterparts [26].

The NLRP3 inflammasome and IL‐1R signaling are essential for *L. amazonensis* elimination, mediated via LTB4 and P2X7 receptors. Chaves et al. [27] demonstrated that deletion of inflammasome components NLRP3, ASC, and CASP1/11 compromised parasite clearance, even in the presence of LTB4 or ATP stimulation. Moreover, CASP11‐deficient mice failed to control parasite loads, underscoring the importance of CASP11 in the host response to *Leishmania* infection [27].

P2X7 receptor activation also triggers mitogen‐activated protein kinases (MAPKs), such as p38MAPK, which regulate NO and ROS production, crucial for the macrophage‐mediated killing of *Leishmania* parasites. Mukherjee et al. [28] described the triterpenoid saponin Spergulin‐A (Sp A) as a potent anti‐*Leishmania* compound that exerts its effects via P2X7 receptor activation. The reduction in parasite burden was detected when Brilliant Blue G (BBG), a P2X7 antagonist, was administered. Additionally, Sp A‐induced P2X7 activation facilitated Ca^2+^ influx, leading to p38MAPK activation, phagolysosome fusion, and enhanced production of NO and ROS, as demonstrated in both *in vitro* and *in vivo* models [28].

El‐Dirany et al. [29] observed that infection with *Leishmania major* downregulated P2X7 receptor expression in skin lesions while inducing the expression of inducible nitric oxide synthase (iNOS), IL‐12, and TNF‐α. Interestingly, IL‐6 levels were significantly elevated in the spleen, suggesting a link between systemic inflammatory responses and P2X7 receptor modulation during infection [29].

P2X7 receptor activation also leads to the release of leukotrienes, particularly LTB4, through the 5‐lipoxygenase (5‐LO) arachidonate pathway, which plays a vital role in the elimination of *L. amazonensis*. Noronha et al. [30] demonstrated that cysteinyl leukotrienes (Cys‐LTs), including LTC4 and LTD4, modulate macrophage responses to *Leishmania* infection. Infected macrophages exhibited reduced Cys‐LT production, implying a protective role for these lipid mediators. Moreover, P2X7‐deficient macrophages produced fewer Cys‐LTs upon ATP stimulation, while exogenous Cys‐LT administration reduced parasite burden and lesion severity in infected mice [30].

The P2X7 receptor is thus integral to host defense mechanisms against *Leishmania* spp., mediating pore formation that facilitates parasite killing. Its role extends to infections with *Toxoplasma gondii*, another intracellular protozoan. Miller et al. [31] demonstrated that P2X7 receptor‐deficient mice are highly susceptible to acute toxoplasmosis. In this context, extracellular ATP functions as a danger signal, promoting NO production via P2X7 receptor‐mediated activation of the transcription factor CREB, which is implicated in regulating nitric oxide synthase expression during *T. gondii* infection [31].

### 3.2. Dengue

Dengue is an arboviral disease transmitted by mosquitoes of the *Aedes* genus and is caused by one of four serotypes of the dengue virus (DENV‐1 to DENV‐4). It is highly prevalent in tropical and subtropical regions and remains the most widespread arbovirus globally. The geographical distribution and disease burden of dengue are projected to increase, largely due to the absence of effective vector control measures and the limited availability of preventive strategies [32].

The P2X7 receptor has been implicated in mediating inflammatory responses during dengue virus infection, particularly in *in vitro* models, where it contributes to the host’s antiviral defense. Several studies have demonstrated that P2X7 receptor activation, through eATP signaling, is closely associated with inflammasome activation during acute viral infections. NS1, a nonstructural viral protein, serves not only as a key virulence factor but also as a diagnostic marker for dengue infection. Corrêa et al. [33] reported that ATP treatment resulted in a significant reduction of NS1 protein levels, suggesting that eATP may enhance the clearance of infected cells during dengue infection [33].

Furthermore, the NO and ROS pathways are closely linked to P2X7 receptor activation, with NO playing a critical role in the host response against various viral pathogens. In their study, Correa et al. observed an increase in NO production following dengue virus infection, which was reversed upon pretreatment with KN62, a selective antagonist of the P2X7 receptor. This observation was corroborated through fluorescence microscopy analysis. Additionally, the study assessed the expression of cytokines and chemokines as markers of host immunomodulation. It was noted that CCL2 and CXCL10 levels were markedly elevated in infected patients; however, ATP treatment during infection reduced the expression levels of these chemokines, indicating a modulatory effect of purinergic signaling on the inflammatory response [33].

### 3.3. Schistosomiasis

Schistosomiasis is an infectious disease caused by three main schistosome species that affect humans: *Schistosoma mansoni*, *S. haematobium*, and *S. japonicum*. It ranks as the second most prevalent parasitic disease globally, accounting for approximately 280,000 deaths per year and affecting an estimated 250 million people across 78 countries [34]. Through bioinformatics analysis, Agboh et al. [35] identified a P2X channel homolog in *Schistosoma mansoni*, with at least four genes encoding P2X receptor‐like sequences in the parasite’s genome. The authors further cloned and expressed the schistosome P2X‐like receptor (schP2X) in *Xenopus oocytes*, enabling functional studies [35].

Once *Schistosoma mansoni* infects the host, its adult worms and eggs interact with the vascular endothelium, releasing vasoactive substances that are critical for regulating vascular tone and homeostasis. NO serves as a major vasodilator produced by the endothelium, capable of inhibiting cytokine‐mediated cellular adhesion and migration. However, schistosomiasis impairs this mechanism by reducing NO synthesis, particularly through the downregulation of endothelial nitric oxide synthase (eNOS) and ATP‐induced NO production in mesenteric vessels [36].

Oliveira, Coutinho‐Silva, and Silva [36] demonstrated that the treatment of mesenteric endothelial cells with BzATP (10 μM) or ATP (3 mM) induced membrane permeabilization, indicating the presence of functional P2X7 receptors. In contrast, endothelial cells derived from P2X7R KO mice (P2X7R^−/−^) did not exhibit this permeabilization in response to the same stimuli. Moreover, activation of P2X7 receptors on endothelial cells by BzATP or ATP stimulated NO synthesis, an effect that was effectively blocked by the selective P2X7 antagonists A740003 and KN‐62 [36]. Additionally, NO production was significantly lower in the infected group compared to the control group upon BzATP stimulation, mirroring the responses observed in P2X7R^−/−^ mice and those treated with antagonists. These findings suggest that infection downregulates functional P2X7 receptor expression in the mesenteric endothelium of affected mice [36].

Transforming growth factor‐beta (TGF‐β) is a key cytokine involved in regulating liver inflammation and promoting host survival during infection. Elevated levels of TGF‐β were detected in peripheral blood mononuclear cells (PBMCs) isolated from *S. mansoni*–infected individuals. Similarly, peritoneal macrophages from infected mice showed increased expression of TGF‐β1. Notably, TGF‐β1 was found to inhibit ATP‐induced permeabilization of macrophages, suggesting a modulatory effect on P2X7 receptor function during infection [37]. Furthermore, peritoneal macrophages from infected mice exhibited reduced ATP‐ and BzATP‐induced permeabilization and Ca^2+^ influx compared to controls. Infected P2X7R^−/−^ mice exhibited a significantly reduced survival rate, highlighting the critical role of P2X7 receptors in host defense mechanisms during schistosomiasis [38].

### 3.4. Leprosy

Leprosy, also referred to as Hansen’s disease, is a curable infectious disease that was declared eliminated as a public health problem by the World Health Organization (WHO) in 2000. Nevertheless, the disease persists as a significant health burden, with approximately 200,000 new cases reported in 2017 and an estimated two million individuals suffering from leprosy‐associated disabilities globally. The WHO classifies leprosy based on the number of skin lesions: single‐lesion leprosy (1 lesion), paucibacillary leprosy (2–5 lesions), and multibacillary leprosy (more than 5 lesions) [39, 40].

The P2X7 receptor exhibits a high degree of genetic polymorphism, particularly single‐nucleotide polymorphisms (SNPs), which may influence host susceptibility to infectious diseases. Souza et al. [41] investigated the association between P2X7R genetic variants (c.1068G > A and c.1513A > C) and leprosy. Through in silico analyses, they identified 61 SNPs within control samples and skin lesion biopsies, among which rs208294, rs1621388, rs7958311, rs1718119, rs1718106, rs3751143, and rs2230911 were the most prevalent. Notably, the presence of the CC genotype of the rs3751143 polymorphism was associated with an increased susceptibility to leprosy when compared to the AA and AC genotypes. Given the established role of the P2X7 receptor in the immune response against *Mycobacterium tuberculosis*, these findings suggest that P2X7R may exert antimycobacterial activity against *Mycobacterium leprae* as well [41].

## 4. Chagas Disease

The progression of Chagas disease involves cellular death and the release of proinflammatory cytokines, such as IL‐1β, both of which are critical for elucidating the immunopathological mechanisms underlying this condition. Among the molecular pathways implicated, the P2X7 receptor has garnered attention as a potential therapeutic target for inflammatory diseases, given its contributory role in disease progression across various infectious contexts [17], thereby suggesting its involvement in Chagas disease pathogenesis.

American trypanosomiasis, commonly known as Chagas disease, is a NTD that predominantly affects populations in Latin America. The etiological agent, *Trypanosoma cruzi*, is transmitted primarily through hematophagous triatomine insects, colloquially referred to as “kissing bugs” [42]. The infection is characterized by an acute phase marked by a pronounced inflammatory response, which often persists into the indeterminate and chronic phases of the disease. These sustained inflammatory processes are closely linked to the parasite’s pathogenic mechanisms, including its capacity for immune evasion, intracellular survival, and proliferation within host tissues [43].

eATP plays a crucial role in modulating host immune responses during infection. ATP stimulation in infected macrophages induces apoptosis, accompanied by the generation of ROS, which are integral to the host’s defense against intracellular pathogens. Notably, insufficient ROS production has been associated with the activation of the NLRP3 inflammasome and subsequent CASP1‐mediated IL‐1β maturation [44]. Despite the recognized importance of purinergic signaling in immune regulation, the specific interactions between purinergic receptors—particularly P2X7—and protozoan infections, including Chagas disease, remain inadequately characterized and warrant further investigation.

### 4.1. Immunopathogenesis and Treatment of Chagas Disease

*Trypanosoma cruzi* is the etiological agent of Chagas disease, a NTD characterized by acute, chronic, or asymptomatic phases. The acute phase presents with nonspecific symptoms, including headaches, fever, and Romanã’s sign, while the chronic phase may remain asymptomatic for years but can progress to severe complications, such as heart failure and hepatosplenomegaly [42].

Transmission occurs primarily through hematophagy by triatomine insects, which excrete infective metacyclic trypomastigotes in their feces during or after feeding. These forms penetrate host cells of the mononuclear phagocyte system, lose their flagellum, and differentiate into amastigotes. Once inside host cells, amastigotes undergo binary fission until the host cell becomes laden with parasites, at which point they transform back into trypomastigotes. The release of these forms into the bloodstream following cell lysis facilitates subsequent infections of new target cells [42]. Transmission routes also include congenital transmission, ingestion of contaminated food, blood transfusion, organ transplantation, and accidental exposure through mucous membranes or broken skin during laboratory procedures [45]. Diagnosis primarily relies on clinical symptomatology during the acute phase [46], although the identification of stage‐specific biomarkers could enhance diagnostic accuracy across the disease spectrum [47].

The parasitic burden during the acute phase of *T. cruzi* infection is a key determinant of host immune activation and influences the progression to chronic disease. Persistent parasitemia drives continuous cellular injury, immune activation, and tissue fibrosis, underscoring the parasite’s pivotal role in disease pathogenesis [17]. During the early stages of infection, macrophages initiate a cytokine cascade by producing IL‐12, which stimulates natural killer (NK) cells to secrete interferon‐gamma (IFN‐γ), TNF‐α, and NO, thereby enhancing parasite clearance. Both M1 and M2 macrophages are activated during the initial phase, contributing distinct immunological functions: M1 macrophages promote proinflammatory responses, while M2 macrophages are involved in tissue repair and immunoregulation [43].

*T. cruzi* invades host cells and utilizes host‐derived nutrients for its metabolic needs. Although the precise energy substrates used during infection remain incompletely understood, studies suggest that glucose, L‐glutamic acid, and L‐proline serve as primary energy sources in the epimastigote stage. However, the metabolic requirements of the metacyclic trypomastigote forms have not been fully elucidated. Notably, epimastigotes differentiate into metacyclic trypomastigotes after approximately 48 h under specific culture conditions [45]. The supplementation of culture media with L‐proline has been shown to sustain parasite viability, indicating its importance as a primary energy source during intracellular stages. Furthermore, ATP depletion in parasites subjected to nutritional stress for 36 h results in diminished motility, highlighting the metabolic vulnerability of *T. cruzi* under such conditions [48].

Extracellular vesicles (EVs) play a crucial role in modulating host–parasite interactions. EVs, which possess a lipid bilayer structure, are secreted into various body fluids and are elevated in the bloodstream during acute and chronic inflammatory conditions, including atherosclerosis, sepsis, diabetes mellitus, stroke, cancer, and preeclampsia [49]. In *T. cruzi* infection, elevated levels of the tumor suppressor protein p53 have been detected in the liver after 45 days, correlating with increased hepatic EV release into the circulation during the acute phase. The administration of 5 mg of *T. cruzi* trypomastigote‐derived EVs (strain Y) to BALB/c mice prior to infection resulted in 100% mortality by Day 22, indicating a significant enhancement of parasite virulence [49].

In chronic Chagas patients, elevated levels of cytokines such as IL‐1β, IL‐7, and IFN‐γ are observed, which are thought to be triggered by host‐derived EVs, including microvesicles and apoptotic bodies. *T. cruzi* expresses surface proteins—such as mucins, gp63, glycosylphosphatidylinositol (GPI)‐anchored proteins, and *trans*‐sialidases—that interact with host immune cells. Additionally, parasite‐derived EVs can induce the secretion of proinflammatory cytokines, including IL‐6, TNF‐α, and NO, via pathways reminiscent of Toll‐like receptor 2 (TLR2) signaling [49].

TLRs are key components of innate and adaptive immunity, recognizing PAMPs and initiating NF‐κB‐dependent transcriptional programs that culminate in cytokine production. Among these, TLR9 has been identified as particularly susceptible to *T. cruzi* DNA, with MyD88‐deficient mice displaying exacerbated parasitemia due to impaired TLR‐mediated signaling. *T. cruzi* stimulates the production of TNF‐α and IL‐12 in a TLR9‐dependent manner, facilitating both innate and adaptive immune responses [50].

Following host cell invasion, *T. cruzi* crosses the vacuolar membrane to replicate in the cytoplasm, engaging intracellular receptors and inflammasomes, including NLRs and IL‐1β‐associated pathways. The activation of NLRP3 and CASP1 upon infection leads to NO production, modulating macrophage function and T‐cell activation [51]. Gonçalves et al. [52] demonstrated that *T. cruzi*–induced IL‐1β production is dependent on NLRP3 and CASP1 activation, with cathepsin B playing a crucial role in this process, as its pharmacological inhibition suppressed IL‐1β secretion.

Benznidazole (BNZ), introduced in the 1960s and 1970s, remains the primary chemotherapeutic agent for Chagas disease. However, its efficacy is limited by adverse effects stemming from nitro reduction and free radical generation [53, 54]. Treatment discontinuation occurs in 6%–40% of patients due to toxicity, despite BNZ’s widespread availability and tolerability in certain populations. Although most adverse effects are reversible or manageable, the interruption of therapy represents a significant challenge to effective treatment [54]. Pontes et al. [55] reported adverse reactions to BNZ, including pruritus, paresthesia, asthenia, and skin rash, with gastrointestinal discomfort and somnolence occurring less frequently. Consequently, the development of novel therapeutic strategies for Chagas disease remains an urgent priority [55].

Chagas disease can also involve the CNS, particularly in chronic stages, leading to cerebrovascular complications such as ischemia resulting from embolic and hypoperfusion events. Left ventricular dysfunction is commonly associated with these neurological manifestations. Despite the absence of evident neuroinflammation, cerebellar and cerebral atrophy have been documented in postmortem analyses of Chagas patients [56]. Chronic Chagas disease is further associated with cognitive deficits, including memory loss, attention impairment, learning difficulties, disorientation, and diminished information processing and reasoning abilities. BNZ treatment has been shown to reduce lipid peroxidation in the cerebral cortex and decrease parasite burden in the CNS. Moreover, BNZ administration during the acute phase of infection prevents depressive‐like behaviors, suggesting a neuroprotective role against *T. cruzi–*induced behavioral alterations [56].

Microvascular platelet–leukocyte aggregates are enhanced during the acute phase, exacerbating cerebral microvasculopathy and promoting leukocyte–endothelium interactions. *T. cruzi* DNA has been detected in the cortex and hippocampus of chronically infected mice, implicating CNS persistence in disease pathogenesis. Furthermore, the CNS remains a critical site for parasite reactivation in immunocompromised individuals, including HIV‐infected patients [56].

A meta‐analysis has identified a polymorphism in the C allele of the P2X7 SNP (rs3751143) as a susceptibility factor for *Mycobacterium tuberculosis* infection. Specifically, individuals carrying the heterozygous AC genotype exhibit a 75% reduction in macrophage‐mediated antimycobacterial activity. Regarding *T. cruzi* infection, polymorphisms such as the P2X7 1513 A/C variant and the rs3751143 C allele have been associated with impaired parasite clearance, potentially contributing to *T. cruzi* virulence and resistance within the host [57].

Chagas cardiomyopathy affects approximately 30% of chronically infected individuals and represents the leading cause of nonischemic cardiomyopathy in Latin America. This form of cardiomyopathy is characterized by focal fibrosis and diffuse myocarditis, predominantly affecting regions such as the left ventricular apex and basal segments of the inferior and posterior walls, leading to ventricular hypertrophy. Sudden cardiac death is the primary cause of mortality in patients with chronic Chagas cardiomyopathy, resulting in a poorer prognosis compared to other etiologies of nonischemic cardiomyopathy. Fatal events are frequently attributed to arrhythmic episodes, including ventricular tachycardia progressing to fibrillation, rupture of left ventricular apical aneurysms, thromboembolic strokes, or progressive global myocardial dysfunction [47].

Electrocardiographic findings indicative of Chagas cardiomyopathy include repolarization abnormalities and multiform premature ventricular contractions, which reflect underlying conduction system impairments such as atrioventricular blocks, sinus node dysfunction, junctional bradycardia, and atrial fibrillation. Comprehensive diagnostic evaluation should incorporate 24‐h Holter monitoring and echocardiography to detect hallmark features of chronic Chagas cardiomyopathy, including mural thrombi, arrhythmias, apical aneurysms, chamber dilatation, and systolic dysfunction [47].

Approximately 5% of patients progress to a severe acute phase, presenting echocardiographic and electrocardiographic abnormalities resulting from fulminant myocarditis, which may culminate in death secondary to congestive heart failure. In untreated individuals, acute infection symptoms typically resolve spontaneously, leading to the onset of the indeterminate phase of Chagas disease. This phase is characterized by the silent invasion of target tissues by *Trypanosoma cruzi*, resulting in subclinical infection and undetectable parasitemia. In the chronic symptomatic phase, dilated cardiomyopathy is the predominant clinical manifestation, accompanied by conduction system abnormalities, including ventricular arrhythmias, left anterior fascicular block, right bundle branch block, and sinus node dysfunction. Structural abnormalities also emerge during this phase, such as secondary embolism and left ventricular aneurysms, which predispose to thrombus formation. Histologically, these lesions are marked by coagulative necrosis of myocytes, hyaline degeneration of muscle fibers, and localized fibrotic replacement [47].

A high prevalence of arrhythmias is a hallmark of patients with chronic Chagas heart disease. Clinically, this is reflected in atrioventricular blocks, bradycardia secondary to sinus node dysfunction, and episodes of ventricular tachycardia. Another distinctive manifestation is the presence of apical lesions, characterized by thinning and deformation of the left ventricular apex with or without thrombus formation. Notably, these lesions differ from arteriosclerotic (postinfarction) aneurysms, as they lack scar tissue formation. In endemic regions, apical lesions are observed in approximately 19% of Chagas patients, with 95% of these cases being associated with electrocardiographic abnormalities such as left anterosuperior divisional block, ventricular extrasystoles, and right bundle branch block. Thromboembolic phenomena are common in patients with Chagas disease, often associated with systemic congestion and cardiomegaly. Autopsy studies have detected thrombosis in 44% of cases, predominantly affecting the apex of the left ventricle and the right atrial appendage. Embolic dissemination to secondary sites, including the kidneys, spleen, lungs, and brain, has also been frequently documented [58].

## 5. Possible Relation Between P2X7 and Chagas Disease

Trypanosomatidae species, particularly *T. cruzi*, express surface membrane proteins known as ectoenzymes, which primarily function to hydrolyze extracellular nucleotides [12]. Among these, ENTPDase hydrolyzes ATP and ADP, which are essential for parasite metabolism, participating in purine salvage pathways crucial for parasite survival. Moreover, ENTPDase activity modulates host cell responses by regulating purinergic receptor activation. Hydrolyzed NTPs and NDPs interact with P2‐type purinergic receptors following enzymatic cleavage by the parasite [12].

ATP, ADP, AMP, and adenosine regulate several cellular processes, including platelet aggregation. ENTPDase and adenosine deaminase (E‐ADA) work synergistically to maintain extracellular ATP homeostasis. The enzymatic activity of ENTPDase and E‐ADA has been implicated in thrombocytopenia and coagulation disturbances during trypanosomiasis [56]. *T. cruzi* expresses a surface‐bound ectonucleotidase identified as Tc‐ENTPDase‐1, a homolog of the CD39 family, which serves as a virulence factor by modulating host immune responses and purinergic signaling [59]. Tc‐ENTPDase‐1 interrupts extracellular ATP‐mediated signaling by inhibiting P2X7 receptor activation, suggesting its dual role as a potential antagonist of P2X7 and as a target for therapeutic intervention in Chagas disease [14]. Furthermore, *T. cruzi* infection elevates serum ATP and adenosine levels in infected hosts, supporting the hypothesis of a functional link between purinergic signaling and disease pathogenesis [60].

Upregulation of NTPDase expression initiates a cascade that increases the activity of other platelet‐associated enzymes, thereby promoting vasoconstriction and impairing platelet aggregation. The resultant reduction of ADP on platelet surfaces contributes to thrombocytopenia, a phenomenon consistently observed in *T. cruzi*–infected models. This dysregulation of homeostatic mechanisms may serve as a protective strategy by mitigating oxidative damage through the suppression of ROS during infection [60, 61].

Elevated E‐ADA activity has been documented in *T. cruzi*–infected mice compared to uninfected controls. Treatments with resveratrol (RSV) and BNZ, whether administered separately or in combination, enhance E‐ADA‐mediated adenosine hydrolysis. This enzymatic activity downregulates adenosine levels, thereby modulating immune responses and reducing astrocyte and microglial activation. Moreover, *T. cruzi* infection upregulates P2X7 and A2A receptor expression in the cerebral cortex, further implicating purinergic signaling in disease progression [62].

ENTPDases, in conjunction with ecto‐5′‐nucleotidase, orchestrate extracellular nucleotide degradation, influencing both P2 and P1 purinergic receptor‐mediated signaling pathways. In Trypanosomatids, such as *T. cruzi* and *Leishmania* species, ENTPDases (TpENTPDases) are vital for parasite nutrition, virulence, infectivity, and adherence to host cells. High virulence is associated with ATP and ADP hydrolysis by ENTPDases, leading to adenosine generation via ecto‐5′‐nucleotidase activity [63].

*T. cruzi* possesses a single ENTPDase gene, TcNTPDase1, whose catalytic activity is Mg^2+^‐dependent and capable of hydrolyzing a variety of nucleotides, including ADP, ATP, UDP, UTP, GDP, and GTP. Conventional ATPase inhibitors fail to suppress this enzymatic activity. However, inhibitors targeting ecto‐NTPDases, such as suramin, ARL67156, and gadolinium, have been shown to reduce both in vivo virulence and in vitro infectivity, highlighting TcNTPDase1’s critical role in parasite infectivity and virulence. This enzyme is localized to intracellular structures—such as the nucleus, kinetoplast, and flagellar insertion site—as well as the external surface of the parasite [63].

Suramin functions as an antagonist of both P2X and P2Y purinergic receptors and serves as a *T. cruzi* ATPase inhibitor. By inhibiting ecto‐NTPDase activity, suramin disrupts purine salvage mechanisms essential for parasite survival and metabolic homeostasis [64]. Given these characteristics, Santos et al. [65] proposed a combinatory therapeutic approach utilizing BNZ and suramin. BNZ reduced parasitemia, inflammation, oxidative stress, and mortality, while suramin induced significant morphological alterations in trypomastigotes, including flagellar detachment, thereby impairing parasite motility and invasiveness. However, suramin’s proinflammatory properties, characterized by elevated IL‐6 levels and the induction of adhesion molecules such as ICAM‐1 and VCAM‐1, raise concerns regarding its potential to exacerbate cardiac tissue damage [65].

*T. cruzi* infection modulates the expression and activity of gap junction proteins, which are integral to intercellular communication and play roles in physiological processes such as cardiac electrical conduction, tissue regeneration, and cellular homeostasis. Gap junction channels permit the passage of small molecules, including ions, glucose, glutamate, ATP, and adenosine [66]. Pannexin‐1 channels facilitate ATP release upon activation by stimuli such as potassium ion flux, hypoxia, or mechanical stress. *T. cruzi* infection enhances pannexin‐1 channel activity, leading to increased extracellular ATP concentrations. This ATP release is essential for *T. cruzi* invasion, as it subsequently activates purinergic receptors [66, 67].

The interplay between pannexin‐1 channels and the P2X7 receptor is of particular interest, as pannexin‐1‐mediated ATP release triggers autocrine and paracrine activation of P2X7 receptors, promoting proinflammatory cytokine secretion, membrane pore formation, and eventual cell death [68]. Cascabulho et al. [69] proposed that extracellular ATP contributes to thymic atrophy during acute *T. cruzi* infection by inducing CD4+/CD8+ thymocyte death via P2X7 receptor activation. In vitro studies have corroborated this relationship, demonstrating ATP‐induced pore formation and solute influx in thymocytes. Although P2X4 receptor activation has been associated with increased membrane permeability to cationic molecules, the specific physiological roles of P2X receptors during infection remain incompletely understood [31].

During thymic atrophy induced by *T. cruzi*, thymocytes exhibit heightened sensitivity to ATP, which induces Ca^2+^ influx and membrane permeabilization via P2X7 receptor activation. This process may involve pannexin‐1 channel upregulation, warranting further investigation into its expression and function in thymocytes. Furthermore, *T. cruzi–*infected peritoneal cells exhibit increased susceptibility to negative regulation of P2X7 receptor activity by extracellular ATP, suggesting complex modulation of receptor responsiveness during infection [31].

A hypothetical mechanism has been proposed wherein *T. cruzi* initiates host cell invasion by mechanically activating pannexin‐1 channels, leading to ATP release, membrane stretching, and depolarization. Elevated extracellular ATP levels sustain infection and promote P2X7 receptor activation, resulting in cytokine release, pore formation, and cell death [31]. Acute *T. cruzi* infection is associated with enhanced hydrolysis of ADP, ATP, and AMP, mediated by upregulated ectoenzymatic activity. The consequent ATP release stimulates the production of cytokines and ROS and RNS, functioning as danger‐associated molecular patterns (DAMPs) that initiate host immune defenses. The proinflammatory response is evidenced by increased serum ATP and ADP concentrations in infected animals, reflecting an immunomodulatory feedback mechanism aimed at controlling parasite proliferation [70]. Despite these findings, the role of the P2X7 receptor as a therapeutic target for Chagas disease remains underexplored, highlighting the need for further studies to elucidate its potential in modulating disease progression and host immune responses.

The role of the P2X7 receptor in modulating both inflammatory and infectious processes supports its candidacy as a therapeutic target for various diseases [71]. Consequently, the pharmaceutical industry and research groups have developed selective P2X7 receptor antagonists. However, these compounds have demonstrated limited efficacy in clinical applications, underscoring the need for the identification of new antagonists capable of effectively treating conditions such as pain and inflammation [72]. Furthermore, the therapeutic potential of P2X7 receptor antagonists, alongside P2X3 receptor antagonists, has been considered for addressing inflammatory disorders, including visceral pain, hypertension, and chronic cough [73]. Therefore, the development of novel treatment strategies for Chagas disease is imperative, with purinergic receptors—particularly the P2X7 subtype—emerging as promising therapeutic targets.

Ergosterol is a sterol essential for parasite replication, playing a critical role in maintaining membrane integrity and function in *Trypanosoma cruzi* [73]. Triazole derivatives have demonstrated potent anti‐*T. cruzi* activity by targeting ergosterol biosynthesis, thereby compromising parasite viability [74]. Among P2X7 receptor antagonists, triazole‐based compounds such as A433977 and A438079 have been developed. Nevertheless, in vivo pharmacological profiles and precise mechanisms of action remain incompletely characterized [72].

Gonzaga et al. synthesized a series of 1,2,3‐triazole derivatives, which exhibited notable biological effects, including anti‐inflammatory and anti‐*T. cruzi* activities. These derivatives effectively inhibited P2X7 receptor‐mediated pore formation in vitro and attenuated inflammatory responses elicited by ATP, carrageenan, or LPS in vivo [71]. Thus, triazole derivatives present a dual therapeutic potential by simultaneously targeting parasitic infection and host inflammatory pathways—both critical components in the pathogenesis of Chagas disease.

## 6. Role of the P2X7 Receptor as a Possible Target for Chagas Disease Treatment

The first evidence suggesting a connection between Chagas disease and the P2X7 receptor demonstrated that this receptor plays a central role in thymocyte depletion and thymic atrophy during *T. cruzi* infection due to its capacity to induce apoptosis, necrosis, and membrane permeabilization [75]. Purinergic signaling is thought to be pivotal in the pathogenesis of Chagas disease, given the sustained elevation of extracellular ATP concentrations during infection. Notably, the activation of P2X7 receptor‐mediated membrane permeabilization is particularly prominent during the acute phase of the disease [76].

### 6.1. Rationale for Targeting P2X7 in Chagas Disease

ATP activates P2 receptors in myocardial tissues, contributing to an increased risk of acute myocardial infarction (AMI) due to its proinflammatory effects, including elevated intracellular calcium levels that promote thromboembolism. Additionally, the upregulation of P2 receptor expression in vascular smooth muscle cells drives vascular growth and remodeling. Among these receptors, P2X7 stands out for its role in amplifying inflammatory responses through the NLRP3 inflammasome and IL‐1β pathways following myocardial infarction, which consequently leads to arrhythmias and sympathetic hyperinnervation. Given these mechanisms, the P2X7 receptor has emerged as a potential therapeutic target for mitigating ischemia–reperfusion injury and may hold relevance in antihypertensive interventions [76].

Pharmacological inhibition of the P2X7 receptor has been shown to attenuate cardiac dysfunction postmyocardial infarction, alleviating conditions such as dilated cardiomyopathy and hypertension by reducing urinary albumin excretion and systemic blood pressure. Moreover, P2X7 receptor activation enhances calcium flux, which promotes thrombosis, facilitates erythrocyte adhesion, and induces platelet aggregation. This receptor’s involvement in prothrombotic pathways underscores its capacity to exacerbate cardiac dysfunction in AMI through thrombogenic mechanisms [76].

### 6.2. Pharmacological Inhibitors and Triazole Derivatives

Genetzakis et al. [77] demonstrated that A74003, a selective antagonist of the P2X7 receptor, significantly reduced infarct size and preserved left ventricular function following occlusion of the left coronary artery in rats, compared to vehicle‐treated controls. Anakinra, an IL‐1 receptor antagonist clinically employed in the treatment of rheumatoid arthritis, also exhibits cardiovascular effects, including modulation of cardiac remodeling, macrophage activation, upregulation of endothelial adhesion molecules, and smooth muscle cell proliferation [77]. In the same study, the combination of A74003 with anakinra, or anakinra alone, did not further reduce infarct size, suggesting that while P2X7 receptor inhibition influences postinfarction cardiac outcomes, it may not fully reverse established dysfunction. Nevertheless, the P2X7 receptor remains a promising therapeutic target for cardiac disease interventions [76].

AMI, characterized by reduced blood supply and sustained ischemia of cardiac tissue, leads to accelerated cardiomyocyte death. Clinical studies have reported upregulated P2X7 receptor mRNA expression in patients with AMI, positioning this receptor as a potential biomarker. Additionally, P2X7 receptor activation exacerbates myocardial injury by enhancing vasopressin activity and elevating ROS levels, as evidenced in animal models. Pharmacological inhibition with BBG attenuated cardiac dysfunction and sympathetic overactivity by suppressing vasopressinergic cell activation and reducing oxidative stress. Furthermore, A438079 attenuated apoptosis, hypertrophy, and fibrosis by inhibiting the PKCβ/ERK signaling pathway. Collectively, these findings underscore the P2X7 receptor’s role in the pathogenesis of various forms of cardiomyopathy and highlight its therapeutic relevance [78].

P2X7 receptor inhibition has also been implicated in modulating atrial electrophysiology and remodeling processes. Its blockade reduces atrial conduction abnormalities, diminishes atrial fibrillation inducibility in sterile pericarditis, attenuates atrial fibrosis, and improves sympathetic regulation, primarily through anti‐inflammatory mechanisms. BBG effectively lowered plasma norepinephrine levels and significantly reduced atrial fibrosis in experimental models of sterile pericarditis. Anakinra was further evaluated for its effects on postoperative atrial fibrillation, demonstrating a reduction in susceptibility to atrial fibrillation induced by sterile pericarditis. These observations identify P2X7 receptor inhibition as a viable strategy for treatment postoperative atrial fibrillation and related cardiovascular disorders [79]. The ATP/P2X7 axis has been shown to play an important role in promoting cardiac hypertrophy and inflammation during pressure overload, as both pharmacological depletion of extracellular ATP and genetic KO of the P2X7 receptor suppressed NLRP3 inflammasome activation in myocardial tissue [80].

During AMI, the assembly of the NLRP3 inflammasome initiates an intense inflammatory response, exacerbating cardiac remodeling, dysfunction, and cell death. P2X7 receptor expression is notably elevated in the border zones of infarcted tissues, a finding corroborated by experiments employing P2X7 antagonists and small interfering RNAs (siRNAs) to inhibit receptor activity. Targeted inhibition of the P2X7 receptor effectively limited infarct size and prevented pathological cardiac enlargement. In rodent models, treatment with BBG or oxidized ATP (oxATP) ameliorated systolic hypertension following myocardial infarction, indicating a significant role of the P2X7 receptor in postinfarction cardiac and neural remodeling. These findings advocate for the exploration of novel pharmacological strategies targeting the P2X7 receptor to improve postmyocardial infarction outcomes [81].

In the context of atherosclerosis, vascular cells are subjected to varying extracellular ATP concentrations due to shear stress and inflammatory insults. The rupture of atherosclerotic plaques is a primary trigger for ischemic stroke and myocardial infarction. Notably, P2X7 receptor expression is markedly lower in nonatherosclerotic arteries compared to carotid arteries harboring atherosclerotic plaques. Experimental studies have shown that genetic deletion of the P2X7 receptor in hypercholesterolemic mice resulted in an approximately 50% reduction in atherosclerotic lesion formation after a 16‐week high‐fat diet regimen. These findings suggest that therapeutic modulation of P2X7 receptor activity or its downstream signaling pathways holds promise for atherosclerosis management [81].

Isoprenaline (isoproterenol), a synthetic *β*‐adrenergic agonist used clinically for bradycardia, can induce cardiac ischemia by disrupting myocardial oxygen balance, leading to excessive extracellular ATP accumulation during ischemia and hypoxia [82]. Experimental models demonstrated that P2X7 receptor KO attenuated isoprenaline‐induced inflammation and myocardial fibrosis, underscoring the receptor’s involvement in cardiac fibrotic processes. Additionally, IL‐1β and IL‐18 levels, along with NLRP3 expression in ventricular tissue, were significantly elevated following isoprenaline administration [83].

Zhang et al. [84] investigated the potential of electroacupuncture at Zusanli (ST36) as an alternative therapeutic intervention for isoprenaline‐induced myocardial fibrosis. Previous research had indicated the anti‐inflammatory effects of ST36 stimulation; however, its impact on isoprenaline‐mediated cardiomyopathy remained unexplored. In P2X7 receptor KO mice, isoprenaline‐induced fibrosis was reversed, accompanied by reduced IL‐1β and IL‐18 levels, as well as downregulation of ventricular NLRP3 and P2X7 receptor expression. These findings implicate the P2X7 receptor as a key mediator of ST36’s antifibrotic effects. Interestingly, while electroacupuncture reduced cytokine levels in P2X7 KO mice, it did not fully prevent hypertrophy or fibrosis, suggesting a complex interplay between P2X7 signaling and alternative inflammatory pathways. In wild‐type mice, ST36 stimulation significantly attenuated isoprenaline‐induced myocardial hypertrophy and fibrosis [84].

Matrix metalloproteinase 9 (MMP9), an enzyme involved in extracellular matrix remodeling, plays a role in physiological processes such as pregnancy‐associated pelvic ligament degradation [85]. Lombardi et al. [86] demonstrated that MMP9 overexpression is driven by macrophage activation during atherosclerosis in murine models. Treatment with A740003, a P2X7 receptor antagonist, effectively modulated MMP9 activity in vascular cells, supporting a mechanistic link between P2X7 receptor signaling and MMP9 regulation via IL‐1β pathways. This observation suggests a potential therapeutic avenue for stabilizing atherosclerotic plaques through P2X7 receptor antagonism [86].

### 6.3. Dual‐Action Potential: Antiparasitic and Immunomodulatory Effects

The indeterminate form of Chagas disease represents a latent stage characterized by the absence of clinical, radiographic, electrocardiographic, and contrast esophagography abnormalities. Standard diagnostic tools, including colon examination, chest radiography, and electrocardiography, fail to detect alterations in this stage. Therefore, more sensitive and sophisticated methods—such as magnetic resonance imaging, myocardial scintigraphy, echocardiography, 24‐h Holter monitoring, cardiopulmonary exercise testing, and noninvasive autonomic assessments—are required to identify subclinical alterations in affected individuals [87].

Histopathologically, focal microscopic inflammatory lesions are present, although parasites are rarely detected by conventional histological methods. Some authors have suggested that these lesions represent a parasite–host equilibrium with limited evolutionary potential. Immunological, enzymatic, and humoral factors, including host genetic polymorphisms, may contribute to variable immune responses, potentially serving as biomarkers to predict disease progression in chronic Chagas disease. Patients with this form generally have a favorable prognosis, with mortality rates comparable to those of uninfected individuals of similar age. However, certain studies have hypothesized a potential risk of sudden death in these patients, although conclusive evidence is lacking. Consequently, patients without digestive symptoms and with normal electrocardiograms are classified as having the indeterminate form or “without apparent cardiomyopathy,” making differentiation from healthy individuals challenging in clinical practice [87].

Progression to the chronic symptomatic phase of Chagas disease is associated with elevated levels of proinflammatory cytokines, including TNF‐α, IL‐7, IL‐10, IL‐2, IP‐10, G‐CSF, MIP‐1*α*, and MCP‐1. A Brazilian study evaluating cytokines and growth factors in chronic Chagas disease patients identified significant alterations in PDGF‐BB, G‐CSF, RANTES, IP‐10, eotaxin, IFN‐γ, IL‐17, IL‐1ra, and IL‐9 levels [88, 89].

Reduced PDGF‐BB levels were implicated in the pathogenesis of Chagas cardiomyopathy, while elevated IP‐10 levels were detected in both indeterminate and symptomatic patients, suggesting its involvement in tissue injury and inflammation. These findings indicate that IP‐10 may serve as a biomarker for disease progression from the indeterminate to the symptomatic cardiac form. Moreover, elevated IL‐10 levels, associated with improved cardiac function, were observed in both indeterminate and symptomatic patients, underscoring the potential of PDGF‐BB and IP‐10 as prognostic biomarkers in chronic Chagas cardiomyopathy [89].

Poveda et al. [90] investigated the profiles of proinflammatory and anti‐inflammatory cytokines in the serum of 109 *T. cruzi*–seropositive individuals, revealing that IL‐1, IL‐12, IFN‐γ, and IL‐6 play crucial roles in Chagas disease pathophysiology and may serve as prognostic markers. IFN‐γ activates macrophages to produce iNOS, TNF‐α, and IL‐1, promoting myocarditis, leukocyte infiltration, cardiomyocyte death, and progressive cardiac inflammation [91]. IL‐4, IL‐10, and TGF‐β downregulate intracellular *T. cruzi* control mechanisms by inhibiting IFN‐γ‐activated macrophages [92]. TNF‐α synergizes with IFN‐γ in controlling intracellular parasites [93], whereas IL‐10 exerts a regulatory role, modulating susceptibility to acute infection [94]. Furthermore, IL‐1β modulates chemokine production and promotes cardiomyocyte hypertrophy during infection [95].

The P2X7 receptor has been implicated in IL‐6 secretion by fibroblasts, contributing to inflammatory processes in conditions such as rheumatoid arthritis and atherosclerosis [96]. The association of cytokines—particularly IL‐1 and IL‐6—with P2X7 receptor activity suggests a possible involvement of this purinergic receptor in the progression of Chagas disease. Upon activation by extracellular ATP, the P2X7 receptor facilitates K^+^ efflux, promoting NLRP3 inflammasome assembly. Consequently, pro‐CASP1 is cleaved into active CASP1, leading to the maturation and release of IL‐1β. Additionally, *T. cruzi* infection induces lysosomal disruption, which further promotes NO secretion and NLRP3 inflammasome activation [52, 97, 98].

**Figure 1:**
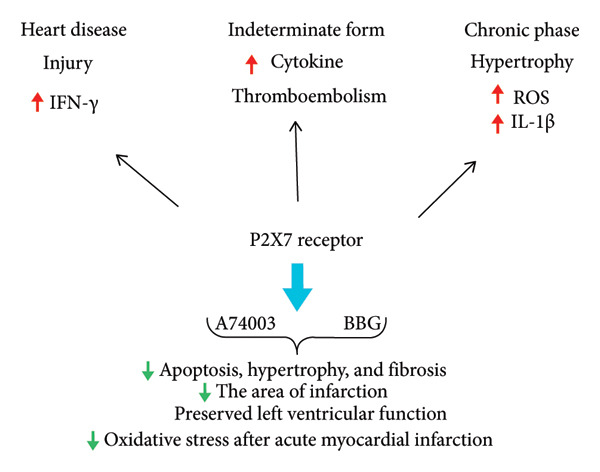
Proposed relationship between the P2X7 receptor and Chagas disease. Extracellular ATP activates P2 receptors in myocardial tissue, initiating proinflammatory signaling cascades. Among these, P2X7 receptor activation plays a critical role by exacerbating cardiac dysfunction through the promotion of thrombosis. In Chagas disease patients, thromboembolisms are frequently associated with systemic congestion and cardiomegaly. The pharmacological inhibition of the P2X7 receptor has been shown to attenuate cardiac dysfunction, reinforcing its involvement in the pathogenesis and suggesting its potential as a therapeutic target for diverse forms of cardiomyopathy. Additionally, P2X7 receptor activation is linked to the release of proinflammatory cytokines and inflammasome activation, providing further evidence of its role in the progression of Chagas disease.

The persistent elevation of proinflammatory cytokines observed during *T. cruzi* infection, coupled with P2X7 receptor‐mediated inflammatory pathways, underscores a potential mechanistic link, as illustrated in Figure 1. A promising therapeutic approach involves the development of triazole derivatives that target both *T. cruzi* and the P2X7 receptor, thereby attenuating parasite burden and mitigating excessive inflammatory responses through inhibition of P2X7‐mediated pore formation. Experimental evidence has demonstrated that P2RX7 gene KO phenotypes exhibit diminished inflammatory responses [99]. Thus, the use of P2RX7 KO cells in experimental models could further elucidate the role of the P2X7 receptor in Chagas disease pathogenesis.

## 7. Knowledge Gaps and Future Directions

Despite significant progress in understanding the role of the P2X7 receptor in inflammation and infection, important gaps remain regarding its involvement in NTDs, particularly Chagas disease. The study of these gaps may indicate innovative treatments that combine antiparasitic and immunomodulatory strategies.

### 7.1. Remaining Uncertainties in the Field

Although preclinical studies have linked P2X7 receptor activation to inflammasome assembly, IL‐1β release, thymocyte loss, and cardiac inflammation in *Trypanosoma cruzi* infection models [69, 75], its precise role in human disease is not clarified. Key aspects, such as the interplay between parasite‐derived ectonucleotidases, host purinergic receptor regulation, and extracellular ATP homeostasis during chronic Chagas cardiomyopathy, are not defined yet. Additionally, the possible relation of CD39/CD73 expression and P2X7 signaling in CD4^+^ T cells may provide essential insights into the host–parasite interaction, which was not explored [100–102].

While in vitro and in vivo studies may suggest correlations between P2X7 activation and proinflammatory responses in Chagas disease, the exact mechanisms by which this receptor modulates disease progression remain uncertain. Key unresolved issues include how different parasite strains interact with purinergic signaling and whether P2X7 would contribute to the transition from indeterminate to symptomatic chronic phases. Moreover, the receptor’s dual role in both pathogen clearance and tissue damage requires a more nuanced understanding in context‐dependent activity. The complexity of P2X7 signaling—including interactions with pannexin‐1 channels, ectonucleotidases, and inflammasome components such as NLRP3—remains insufficiently characterized in humans. Supporting this, P2X7‐KO mice infected with *T. cruzi* exhibit altered cardiac cytokine profiles, with decreased IL‐6 and IFN‐γ and increased IL‐10 and IL‐12p70, suggesting that purinergic modulation varies depending on both host and parasite context [103].

### 7.2. Need for Further Clinical and Translational Studies

A preliminary investigation reported P2X7 expression in lymphocytes from individuals with the indeterminate form of Chagas disease; however, its clinical implications are yet to be clarified [104]. Systematic reviews have underscored enduring gaps in both diagnostics and therapeutics, including heterogeneity in study design, variability in outcome measures, and insufficient follow‐up durations [100]. Addressing these limitations will require well‐structured longitudinal cohorts and early‐phase clinical trials to rigorously evaluate P2X7 as both a biomarker and a therapeutic target.

To date, no longitudinal human studies have directly assessed P2X7 expression or activity across the different stages of Chagas disease, nor clarified the influence of P2RX7 genetic polymorphisms on disease susceptibility or therapeutic response. This absence extends to clinical trials in which no studies have tested P2X7 antagonists—such as triazole derivatives—in patients with Chagas disease. These gaps underscore the need for pharmacogenetic studies and rigorously designed therapeutic trials to associate preclinical discoveries and clinical application [87, 105, 106].

### 7.3. Suggestions for Novel Approaches

Future research on the P2X7 receptor in Chagas disease should integrate therapeutic innovation with advanced methodological strategies, involving the development of dual‐action molecules, such as triazole derivatives, which combine antiparasitic activity against *T. cruzi* with inhibition of P2X7‐mediated pore formation, thereby attenuating inflammation [71, 74]. Drug repurposing also represents an interesting strategy, since P2X7 antagonists already evaluated in inflammatory diseases such as rheumatoid arthritis could be repositioned for Chagas disease, potentially shortening development timelines and costs [77].

At the same time, cutting‐edge immunological tools—including single‐cell transcriptomics, CRISPR‐Cas9 gene editing, and organ‐on‐chip platforms—may provide insights into the cell type–specific and tissue‐level roles of P2X7 signaling during infection [107, 108]. For instance, a human malaria‐on‐a‐chip model has been developed to simulate the full lifecycle of *Plasmodium falciparum* and assess the efficacy of antimalarial drugs [109].

Additional strategies include the development of biomarkers based on P2X7 receptor activity—such as inflammation‐ or fibrosis‐associated markers evaluated in the progression from indeterminate to cardiac forms of Chagas disease—the use of nanotechnology‐based delivery systems to enhance drug bioavailability in target tissues, including noninvasive approaches capable of crossing the blood–brain barrier, and the application of advanced imaging techniques, such as PET tracers for P2X7, to map receptor expression and inflammation in real time [110–112].

Finally, sustained interdisciplinary collaboration will be essential. Platforms such as the DNDi Chagas Clinical Research Platform already offer valuable frameworks for multistakeholder partnerships, fostering research that bridges immunology, pharmacology, molecular biology, and clinical medicine [113, 114]. By combining these approaches, the study with P2X7R may not only be a contributor to the study of Chagas pathogenesis but also as a biomarker and therapeutic target capable of transforming patient care.

## 8. Conclusion

Given the increasing recognition of purinergic signaling in host–pathogen interactions, particularly the role of the P2X7 receptor, further investigation of its involvement in Chagas disease is extremely necessary.

Evidence indicates a contribution of P2X7 to the pathophysiology of *Trypanosoma cruzi* infection, concerning immune regulation, disease progression, and cardiac pathology. Nevertheless, critical gaps remain regarding their precise molecular mechanisms, biomarker potential, and clinical applicability.

Addressing these gaps will require comprehensive research, which integrates basic, translational, and clinical studies. By validating P2X7 as both a critical role in the disease and an able target therapeutic point, it may advance toward strategies that improve treatments for individuals affected by this NTD.

## Conflicts of Interest

The authors declare no conflicts of interest.

## Author Contributions

Caroline de Souza Ferreira Pereira: conceptualization, methodology, and writing–original draft preparation. Robson Xavier Faria: visualization, supervision, and writing–reviewing and editing.

## Funding

This study was funded by Conselho Nacional de Desenvolvimento Científico e Tecnológico, 316568/2021‐0; Coordenação de Aperfeiçoamento de Pessoal de Nível Superior, 001; and Fundação Carlos Chagas Filho de Amparo à Pesquisa do Estado do Rio de Janeiro, E‐26/203.246/2017 E‐26/211.025/2019 E‐26/200.982/2021.

## Data Availability

The data that support the findings of this study are available from the corresponding author upon reasonable request.
